# Effects of zinc ex vivo on taurine uptake in goldfish retinal cells

**DOI:** 10.1186/1423-0127-17-S1-S13

**Published:** 2010-08-24

**Authors:** Sonia Nusetti, Mary Urbina, Lucimey Lima

**Affiliations:** 1 Laboratorio de Neuroquímica, Centro de Biofísica y Bioquímica, Instituto Venezolano de Investigaciones Científicas, Caracas, Venezuela

## Abstract

**Background:**

Taurine and zinc exert neurotrophic effects in the central nervous system. Current studies demonstrate that Na^+^/Cl^-^ dependent neurotransmitter transporters, similar to that of taurine, are modulated by micromolar concentrations of zinc. This study examined the effect of zinc sulfate *ex vivo* on [^3^H]taurine transport in goldfish retina.

**Methods:**

Isolated cells were incubated in Ringer with zinc (0.1–100 µM). Taurine transport was done with 50 nM [^3^H]taurine or by isotopic dilution with taurine (0.001–1 mM) and 50 nM [^3^H]taurine.

**Results:**

Zinc reduced the capacity of taurine transport without changes in affinity, and caused a noncompetitive inhibition of high affinity taurine transport, with an EC_50_= 0.072 µM. The mechanism by which zinc affects taurine transport is unknown at the present.

**Conclusions:**

There may be a binding site of zinc in the transporter that affects union or translocation of taurine, or possibly the formation of taurine-zinc complexes, rather than free zinc, could affect the operation of the transporter.

## Background

Taurine (2-aminoethane sulfonic acid), a β-amino acid that contains a negatively charged sulfonic acid group, is present at high levels in the retina of many vertebrates [1]. This amino acid is known to be involved in the mediation of multiple functions, such as osmoregulation, modulation of calcium fluxes, neuromodulation, protection from oxidative stress, modification of protein phosphorylation, membrane stabilization, affectation of cell migration in the brain and in the retina, regulation of axonal outgrowth, elevation in the number of regenerating retinal cells after nerve lesion, and production of neural protection in certain neuropathies [2-4]. Thus, taurine possesses neuroprotective and neurotrophic properties in the central nervous system (CNS) during development and regeneration. Some taurine functions are similar to those of zinc, an element with relevance in metabolic, genetic and neurotrofic processes [5,6].

Zinc is highly concentrated in the retina [7]. The physiological functions of zinc have been studied in retina, where it is believed to interact with taurine, to modify photoreceptor plasma membranes, to modulate synaptic transmission and to serve as an antioxidant [8,9]. Zinc is essential for the normal development and function of the CNS and a deficiency of this element during early development can result in gross structural defects and pronounced behavioural abnormalities [10,11]. Experimental evidence demonstrates that zinc can have both positive and negative effects on the cells, depending on the local concentration or state of the cell. An excess of free zinc can be harmful and trigger *in vitro* as well as *ex vivo* neuronal damage [12-14]. The neuroprotective effect of low concentrations of zinc in the brain has been attributed to its antioxidant function and to the fact that zinc is an important modulator of excitatory synaptic transmission [7,15]. Zinc occurrence in cultures of goldfish retinal fragments at a concentration equal to or greater than 0.06 µM in the presence of taurine reduces the amino acid trophic effect, which could be related to the interaction between both in the membranes or to the influence of zinc on taurine transport [16]. It has been demonstrated that micromolar concentrations of zinc modulate the activity of voltage-dependent calcium channels, of receptors and of neurotransmitter transporters [17-20]. Taurine transporter, together with dopamine, norepinephrine and serotonin transporters, modulated by zinc, form a part of a subfamily within the large family of the Na^+^/Cl^-^-dependent transporters, all of which share the same topology of 12 transmembrane domains [21]. The trophic effect of taurine may be affected by the inhibition of its transport [22]; given that zinc, in concentrations equal to or greater than 0.06 µM, blocks the trophic function of this amino acid, that the effect of taurine could be influenced by transport inhibition, and that zinc modulates neurotransmitter transporters with the same topology, the evaluation of whether the presence or absence of zinc could affect taurine uptake was proposed as the objective of this work.

## Methods

### Animals and preparation of cell suspension

Goldfish (*Carassius auratus*), measuring 3–4 cm, from a local commercial breeder (Fauna Roosevelt, Caracas, Venezuela), were kept in an aquarium in the Laboratory under 12:12-h light cycle for 1–3 weeks before use. The fish were dark adapted for 30 min, which produces retraction of the retinal pigmented epithelium from the retina, and anesthetized with tricaine (0.05%) prior to enucleation of the eye. The eyes were rinsed in Locke’s solution free of Ca^2+^ and Mg^2+^ and the retina was dissected, placed in the same solution with trypsin and incubated at 25^0^C for 30 min. The weight per retina was 30–35 mg and contained 1–2 mg of total proteins. Mechanical dissociation was performed gently with a Pasteur pipette. After centrifugation at room temperature with a swinging rotor in a Damon/IEC Division centrifuge at 600 g for 5 min, the pellet was washed and finally resuspended in Ringer solution (NaCl 125 mM, KCl 5 mM, CaCl_2_ 2 mM, MgSO_4_^.^7H_2_O 1.3 mM, Na_2_HPO_4_^.^2H_2_O 0.4 mM, NaH_2_PO_4_ 0.1 mM, NaHCO_3_ 25 mM, glucose 10 mM) [23].

### Kinetics of transport

Initially, the uptake of 100 nM [^3^H]taurine (891.7 GBq/mmol) into total retinal cells was measured in a preparation of approximately 250,000 cells per tube for different periods of time: 0.5, 2, 4, and 10 min to confirm the linearity of the uptake process. Also uptake of 100 nM [^3^H]taurine was determined in dilutions of cells preparations of 100, 200, 300 and 500,000 cells per test tube. The cell preparation was preincubated at 25°C for 2 min in Ringer solution. Uptake was initiated by adding 100 µl of uptake buffer containing radiolabel substrate. After incubation, the process was stopped by rapid filtration through fibreglass filters (Watman GF/B), followed by two washes with 5 ml of cold Ringer solution. The filters were placed in scintillation vials, dried, and counted in 4 ml of toluene/omnifluor 0.04% in a Packard scintillation counter model Tricarb 1900 TR (efficiency 65-67%) [23].

### Saturation assays

The uptake of taurine was performed by isotopic dilution experiments with 50 nM [^3^H]taurine and increasing concentrations of taurine from 0.001 to 1 mM, and in a preparation of about 250,000 cells per test tube to explore the high and low affinity component of the carrier. The transport was sodium dependent, and 80-90% inhibited by hypotaurine and beta-alanine (50 µM), but not by gamma-aminobutyric acid (GABA). Finally, saturation assays were measured with a concentration of taurine to target the high-affinity taurine uptake (0.01 to 1 µM) [23]. The cell preparation was incubated in the presence of various concentrations of [^3^H]taurine from 0.01, 0.025 and 0.05 µM, followed by isotopic dilutions with concentrations of taurine from 0.05 to 1 µM and 50 nM [^3^H]taurine. The preparation was preincubated at 25°C for 2 min in Ringer solution. Incubation was started by the addition of the substrate. After 5 min the process was stopped by filtration. The experiments were performed in triplicates.

### Effect of zinc *ex vivo* on [^3^H]taurine transport

The effect of zinc sulphate (ZnSO_4_) was studied by preincubating the cell preparation with different concentrations of zinc, from 0.001 to 500 µM. The uptake was initiated by the addition of 50 nM [^3^H]taurine plus 50 nM cold taurine. After 5 min of incubation at 25°C, the cells were washed and the process was stopped by filtration. This experiment was done to determine the mean effective concentration (EC_50_) of ZnSO_4_. The mechanism by which ZnSO_4_ affects taurine uptake was explored by high affinity [^3^H]taurine uptake in the presence and in the absence of different concentrations of ZnSO_4_ (0.1, 1, 50, 100 µM).

### Statistical analysis

Non-linear fitting was done with the program PRISMA (GraphPad Prism 2). V_max_ and K_s_ of taurine transport were calculated either by Lineweaver-Burk plots or by curvilinear analysis. EC_50_ of ZnSO_4_ was determined by curvilinear analysis. Each value is expressed as mean ± standard error of the mean (SEM). The probabilities of the differences among means were derived from the analysis of variance (ANOVA), INSTAT 2 program [24]. Significance was considered if p < 0.05.

## Results

### Cell preparation and incubation time

[^3^H]Taurine uptake was linear respecting the number of cells between 50,000 and 400,000 per tube. For all experiments 250,000 cells were used. The uptake was linear during 10 min of incubation at 25°C. The incubation was fixed at 5 min for the rest of the analysis.

### Kinetic parameters of [^3^H]taurine transport

**Figure 1:**
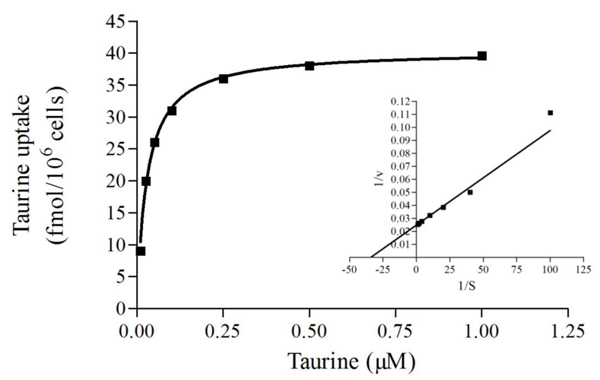
**Representative curve of the saturation of [^3^H]taurine uptake into goldfish retinal cells determined by isotopic dilution.** Data are best fitted to a rectangular hyperbola R^2^=0.96. The insert correspond to Lineweaver-Burk analysis of the same results, R^2^=0.98.

The analysis of saturation experiments performed in the presence of 50 nM [^3^H]taurine alone and with increasing concentration of non labeled taurine from 0.001 to 1 mM resulted in a best fitting to a two site model. The kinetic parameters of the high affinity component were K_s1_ = 0.02 ± 0.003 µM and V_max1_ = 34.36 ± 0.31 fmol/l/10^6^ cells, and for low affinity component, were K_s2_ = 5.29 ± 0.110 µM and V_max2_ = 55.50 ± 1.29 fmol/l/10^6^ cells. To work with the high affinity component [23], taurine uptake was measured in 0.01-1 µM concentration range. The resulting saturation curve was hyperbolic, according to the Michaelis-Menten kinetic model (Figure 1). Data obtained were transformed using the double reciprocal method, producing a linear graph with R^2^ = 0.94; this was utilized to determine the kinetic parameters of the transporter, K_s_= 0.03 ± 0.005 µM and V_max_ = 38.72 ± 2.29 fmol/10^6^ cells.

### Effect of zinc on [^3^H]taurine transport

**Figure 2:**
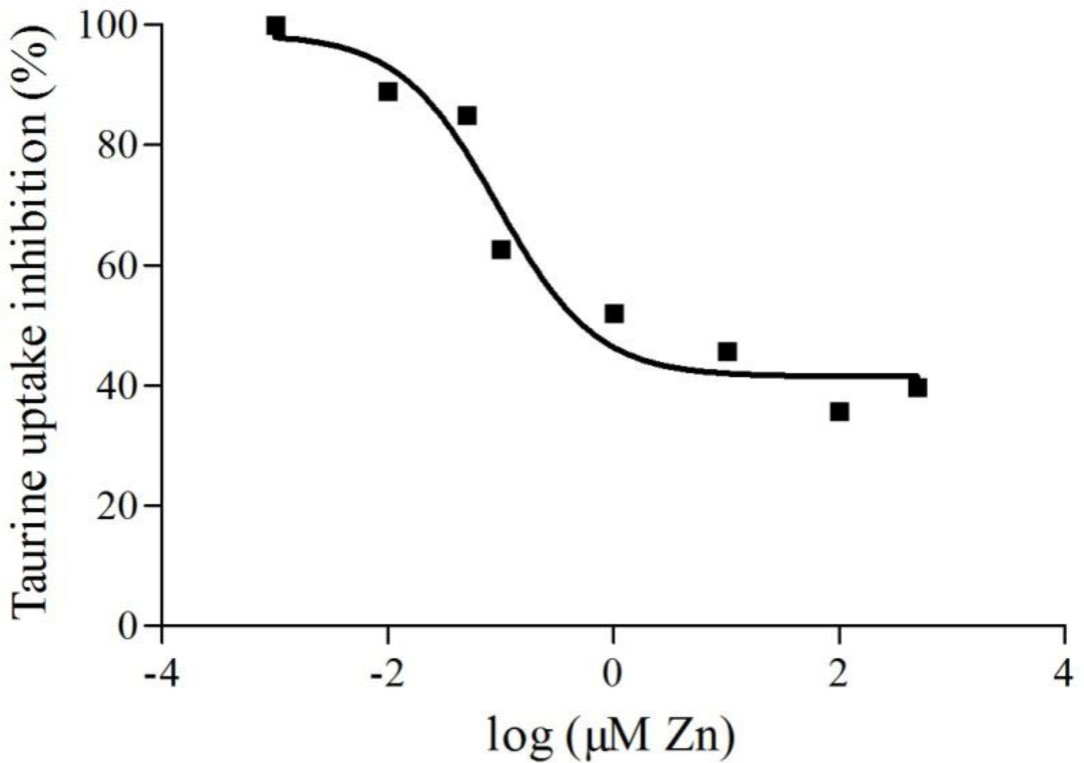
Representative curve of the zinc inhibition on [^3^H]taurine uptake (%) in goldfish retinal cells.

**Figure 3:**
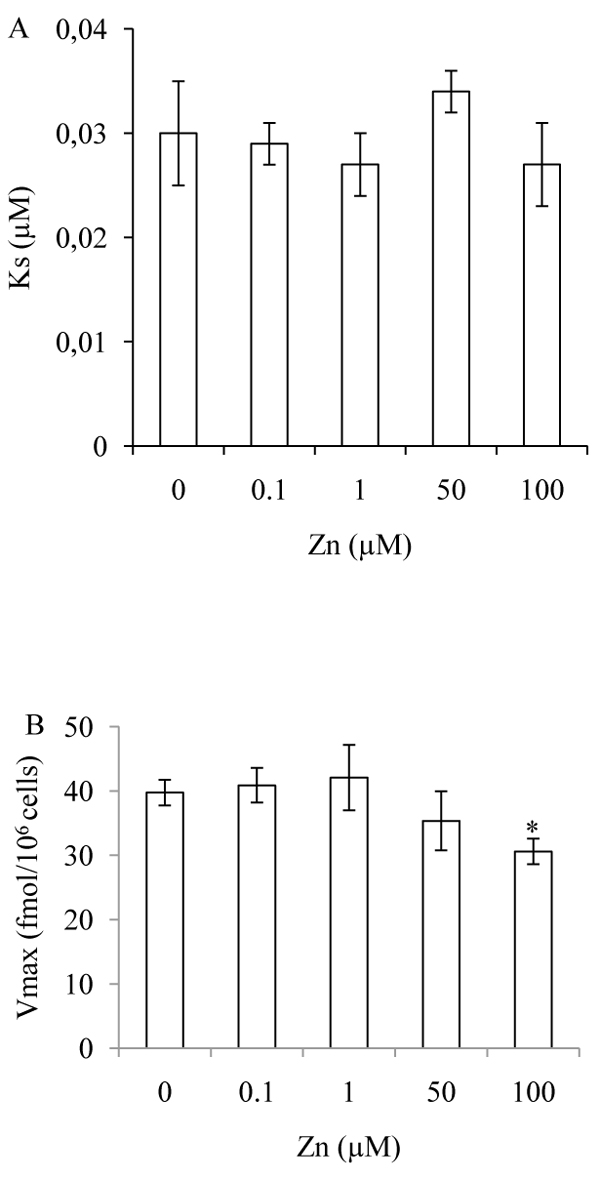
**Saturation experiments of high affinity of [^3^H]taurine uptake, Ks (A) and Vmax (B), in the presence of variable concentrations of zinc.** Each value is the mean ± SEM, n = 3. * p < 0,05 with respect to control.

The simultaneous incubation of cell preparation with ZnSO_4_ in concentrations ranging from 0.001 to 500 µM reduced the uptake with an EC_50_= 0.72 ± 0.013 µM (Figure 2). The mechanism by which the zinc affected high affinity [^3^H]taurine uptake was explored, at a zinc concentration of 100 µM, a 30% reduction in taurine transport capacity was observed, V_max_= 30.58 ± 2.10 fmol/10^6^ cells, compared to the control group, V_max_= 39.72 ± 2.02 fmol/10^6^ cells. At concentrations of 0.1, 1 and 50 µM of zinc there were no significant modifications in either the V_max_ or K_s_ of taurine transport as compared to the control group (Figure 3).

## Discussion

### Effect of zinc on taurine transport in isolated cells from goldfish retina

Taurine transport in isolated cells from goldfish retina presented two components, one of high and another of low affinity, results that agree with those obtained by Guerra *et al.*[23] and Lima *et al*. [25]. The presence of two components of taurine transport, high and low affinity, has been demonstrated in cells isolated from rat retinas [4], in pigment epithelial cells from both rats [26] and humans [27], and in human lymphocytes [28]. In the present study, zinc caused a noncompetitive inhibition of high affinity taurine transport, with an EC_50_= 0.072 µM. Taurine transport inhibition did not reach 50% with the concentrations used, probably reflecting the variable sensitivity of the components or the limited sites of zinc for exerting its effect. It might be that higher concentrations of zinc are needed to cause a greater inhibition, although the shape of the curve showed a flatness at the higher concentrations tested. It may also be that greater inhibition was not observed because zinc affects only one of the taurine transporters, TAUT-1 or TAUT-2, and the unaffected transporter maintains the uptake. The mechanism by which zinc affects taurine transport has not been demonstrated. There may be a binding of zinc in the transporter that affects union or translocation of taurine, or possibly the formation of taurine-zinc complexes, rather than free zinc could modulate the operation of the transporter. In fact, taurine, taurine transporter TAUT-1, and zinc coexist in ganglionic cells and in photoreceptors of goldfish and rat retinas [29].

Recent work has demonstrated that zinc also modulates Na^+^/Cl^-^-dependent neurotransmitter transporters, such as those of monoamines [19,20] and other neurotransmitter transporters as glutamate, histamine and gamma-aminobutyric acid (GABA) [30-32]. Most of these studies coincide in that the effect of zinc, whether stimulation or inhibition, is produced directly on the transporter. Zinc enhances the binding of cocaine analogues to dopamine transporter, and behaves as a noncompetitive inhibitor of dopamine transport in synaptosomal membranes, 10 µM zinc inhibits V_max_ by 30% and 1 mM by 50%), an effect due to the union of zinc in an area of the transporter that affects the translocation of the monoamine [33]. Residues of the proteins that interact with zinc, His^193^, His^375^, and Glu^396^, which are close to one another, have been recognized in the tertiary structure of this transporter, and zinc facilitates the formation of oligomeric complexes of the transporter that affect the process of translocation of the neurotransmitter [21,34,35].

It has also been reported that zinc inhibits the transport of glutamate in Müller cells and retinal cones of salamanders with an IC_50_=0.84 µM [36]. The effect of zinc was rapid and irreversible, which suggests a direct effect on the inhibition of transport, but the ion does not compete for the binding sites of glutamate, sodium, potassium or for transporter protons, but instead binds to an external point to the membrane, which implies an allosteric modulation transporter function [36]. Glutamate EAAT1 transporter expressed in oocytes of *Xenopus laevis* is inhibited by zinc, with an IC_50_=9.9 µM, and on the basis of studies by site-directed mutagenesis it has been reported that His^146^ and His^156^ residues of EAATI form part of the binding sites of zinc. These residues are found on the extracellular edge of the third transmembrane domain. Zinc bond alters the shape of this region, which reconstructs the contour of the pore, thus regulating the passage of the substrate and ions through the transporter [30].

Zinc reduced taurine transport in goldfish retinas with an EC_50_ = 0.072 µM. An ample range of effective concentrations of zinc has been reported regarding its effect on voltage-dependent calcium channels, with an IC_50_ = 69 µM, for all dopamine and glutamate transporters, with an EC_50_ = 0.79 and 0.84 µM respectively, and for *N*-methyl *D*-aspartate (NMDA) and GABA receptors, with EC_50_ of 0.1-100 µM and 0.6-320 µM respectively [17,33,36-38]. The concentration of extracellular zinc estimated in the brain at rest is 10 to 20 nM [39]; however, the concentration of total zinc in goldfish retina is 413 nM [40], and under these conditions zinc does not affect taurine transport rate. As well as in other areas of the CNS, the labile zinc of the retina is released during neuronal activity, although it is possible that taurine transport could be inhibited when there is an excess of extracellular zinc, resulting in neurodegeneration. Culture retinal cells exposed to 300-500 µM of zinc during 15 min results in significant neuronal death [41].

The trophic effect of taurine is inhibited by µM concentrations of zinc in cultures of goldfish retinal explants [16]. In addition, taurine does not counteract the inhibitory effect of 1 µM zinc [16], which may be due to inhibition of taurine transport. This conclusion is consistent with the fact that the trophic effect of the taurine is dependent on its intracellular levels [42,43]. Zinc deficiency alters taurine levels in goldfish retina, its trophic effect and increases taurine uptake [40]. Zinc *ex vivo* and zinc deficiency have different effects on taurine transport, and the mechanisms on this transport remain to be clarified.

## Conclusions

Zinc reduced taurine transport capacity and noncompetitively inhibited it. The mechanism by which zinc affects taurine transport is unknown at the present. There may be a binding site of zinc in the transporter that affects the union or the translocation of taurine, or probably the formation of taurine-zinc complexes, rather than free zinc, modulates the operation of the transporter.

## Abbreviations

Mean effective concentrations (EC_50_), Constant affinity (Ks), Max capacity (Vmax), standard error of the mean (SEM) and taurine transporter (TAUT).

## Competing interests

The authors have non-financial competing interests in an exclusive academic way.

## Authors' contributions

SN discussed the design, carried out the experiments, made calculations, participated in the discussion of results, and did most of the writing. MU actively participated in transport experiments, in standardizing conditions, commenting results, and in sustaining the basic facilities for performing accurate experiments. LL is the Grant recipient, made the contribution for conception, design, and analysis, interpretation of data, discussion, and final writing.
